# Different Surface Electromyography of Propagated Sensation along Meridians Produced by Acupuncturing Quchi Acupoint (LI11) or Control Points

**DOI:** 10.1155/2013/198451

**Published:** 2013-09-03

**Authors:** Chun-Ri Li, Yifan Lin, Hong-Yang Guan, Zhe-Rui Liang, Zhi-Xing Zhang, Andre Kim, Jong-Myung Ha, Lu Ren, Peijing Rong, Zhong-Yue Gu, Yi-Guo Chen

**Affiliations:** ^1^Liaoning University of Traditional Chinese Medicine, 79 Chongshan East Road, Huanggu District, Shenyang 110847, China; ^2^Department of Traditional Chinese Medicine, General Hospital of Shenyang Military Region, Liaoning, China; ^3^Department of Pharmaceutical Engineering, College of Medical Life Sciences, Silla University, Busan 617-736, Republic of Korea; ^4^Institute of Acupuncture and Moxibustion, China Academy of Chinese Medical Sciences, Beijing 100700, China

## Abstract

This study investigated the propagated sensation along meridians (PSM) produced respectively by acupuncture at a specific acupoint of right-side Quchi (LI11), a nonacupoint on meridian (control meridian point), and neither meridian nor acupoint (control point). All the stimulated points were on the right brachioradialis along the large intestine meridian of hand Yangming. Surface electromyography (sEMG) was used to reflect the activity of the brachioradialis along the large intestine meridian of hand Yangming. The PSM rate of LI11 (59.21%) and the control meridian point (53.95%) were significantly higher than the control point (38.16%) (*P* < 0.05). After acupuncture, the brachioradialis sEMG amplitude was 5.08 ± 2.93 uV at LI11, 3.08 ± 1.18 uV at the control point, and 2.77 ± 1.36 uV at the control meridian point. The amplitude of LI11 was significantly higher than both the control meridian point and the control point (*P* < 0.05). When the sEMG activity of brachioradialis returned to the stable base line, brachioradialis sEMG duration at LI11 (265 ± 87.87 s) was significantly longer than that at the control meridian point (91.69 ± 42.98 s) and the control point (83.31 ± 32.76 s) (*P* < 0.05). In conclusion, acupuncture activated PSM at all points but showed an acupoint specificity at LI11 and a meridian specificity at the control meridian point.

## 1. Introduction 

According to the Spiritual Pivot: Nine Needles and Twelve Source Points, the key effect of acupuncture is the arrival of Qi which possesses significant curative effects. The arrival of Qi not only contained Deqi but also comprised propagated sensation along meridians (PSM), which played an essential part in acupuncture clinical efficacy [1]. PSM has been studied for many years, and it has been reported with numerous pieces of evidence as a kind of conduction phenomenon along classic meridians that may be accompanied by sensations of Deqi such as soreness, numbness, heaviness, and distention [2–4]. On the limbs, PSM was generally accordant with the running route of classic meridians. The essence of PSM might be characterized by soreness, numbness, distention, twitching, cold, or heat. Differing from nerve conduction, PSM is bidirectional and much slower in speed. On the limbs, the scope of PSM is about 0.2 to 2.0 cm in width [5]. Moreover, its conduction is not constant and has pause points at certain acupoints. The phenomenon of PSM was different from Deqi, which was completely the subjective feeling. Recent experimental research about PSM has focused on muscles, blood flow, and nerves [6, 7].

Based on the consistency between PSM and the classic meridians on the limbs, this study aimed at exploring surface electromyography (sEMG) activity variations of brachioradialis along the large intestine meridian of hand Yangming after acupuncturing at LI11, the control meridian point, and the control point in every subject.

## 2. Materials and Methods

### 2.1. Subjects

The subjects of this study consisted of 76 cases of healthy volunteers (39 males, 37 females, age 20.2 ± 2.32 years, weight 55.15 ± 10.34 kg, and height 167.3 ± 9.5 cm) who were students at the Shenyang Institute of Physical Education. Prior to participating in the study, all subjects were informed of the trial protocol, and they consented to participate in this study. The protocol was approved by the Ethics Committee of the Affiliated Hospital of Liaoning University of Traditional Chinese Medicine (2011KT-009). The subjects had no history of tobacco and alcohol use and all were right handed. The subjects had no experience with acupuncture. They were screened and excluded for major medical illnesses and history of injuries to arms, such as scars, fracture, dislocation, and nerve injury.

### 2.2. Acupuncturing Strategies

#### 2.2.1. Selection of Needling Points

**Figure 1:**
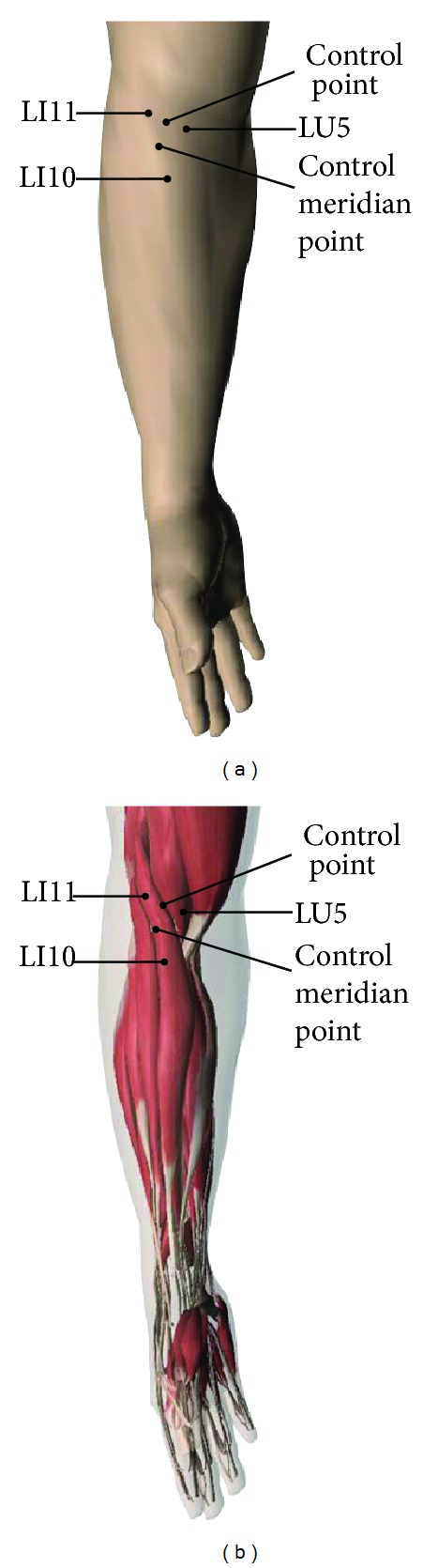
Marked points to be acupunctured. Quchi (LI11), nonacupoint on meridian (control meridian point), neither meridian nor acupoint (control point), Shousanli (LI10), and Chize (LU5).

Three stimulating points were acupunctured respectively, including LI11, a nonacupoint on meridian (control meridian point), and neither meridian nor acupoint (control point) on the right side. The control meridian point was located between LI11 and Shousanli (LI10) on the large intestine meridian of hand Yangming, and the control point was located between LI11 and Chize (LU5) on the brachioradialis (Figure 1). All points were on brachioradialis. Acupoints were located by referring to the “Standard Project of Acupoint” issued by China in 2008 [8].

#### 2.2.2. Methods of Acupuncture

**Figure 2:**
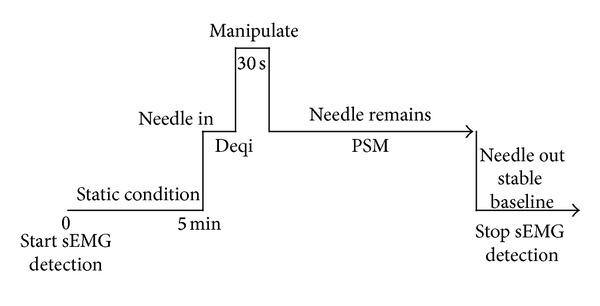
Flow diagram of acupuncture and sEMG detection.

All acupuncture was performed by the same acupuncturist, who had 30 years of clinical experience. “Hua Tuo” acupuncture needles (0.35 × 40 mm) were used at all stimulation points. Every subject was acupunctured at LI11, the control meridian point, and the control point, and the stimulation of every point was performed at intervals of one day. The points were acupunctured perpendicularly, and the acupuncturist manipulated by lifting, thrusting, and rotating until Deqi. After Deqi, the acupuncturist continued to manipulate for 30 s to activate PSM. The rotation of the needle was approximately 180° clockwise and anticlockwise with a combination of lifting and thrusting at the rate of 60 times/min. When the conduction of PSM reached the wrist on the right side, the data was considered as PSM, and the needle was left in the point until the myoelectricity of brachioradialis returned to the stable baseline (Figure 2). When the subjects felt pain close to the pain threshold during the acupuncture stimulation, the acupuncturist promptly altered the needling methods. Once PSM was activated, acupuncture was not continued. If the acupuncture was continued for three times without activating PSM, the stimulation was considered as a failure of PSM activation, and the data were not included in the statistical analysis of sEMG amplitude and duration.

#### 2.2.3. Steps of Acupuncture and sEMG Detection

The sEMG activity was recorded telemetrically using an eight-channel sEMG system (ME6000, Mega Electronics Ltd., Kuopio, Finland). To maintain a low degree of the interelectrode resistance (<2 kΩ), the skin was shaved, rubbed with sandpaper, and then cleaned with a 75% alcohol solution. Electrodes with an interelectrode distance of 20 mm were then positioned longitudinally along the brachioradialis.

Firstly, sEMG of the brachioradialis was detected under a static condition for 5 min. After sEMG baseline became stable, LI11, the control meridian point, and the control point on the right side were acupunctured respectively with an interval of one day. Once PSM was activated, sEMG was detected until it returned to the stable baseline.

### 2.3. Data Analysis

The sEMG signals with a sample of 1000 Hz were inputted in MegaWin software. The sEMG data were filtered by 10–500 Hz and then were fully wave rectified by root mean square (RMS) with a frame width of 50 s. The time of sEMG activity was defined as the sEMG duration when the EMG activity of the muscle exceeded its mean from the static condition by three standard deviations [9]. The myoelectric signal amplitude was defined with a frame width of 50 s of initial sEMG activity.

The data of positive PSM were classified into static group, LI11 group, control meridian point group, and control point group. Statistical analysis was performed using SPSS software (version 11.0, SPSS Inc., Chicago, IL, USA). *P* value less than 0.05 was considered as a significant difference.

## 3. Results

### 3.1. Comparison of PSM Rate

**Figure 3:**
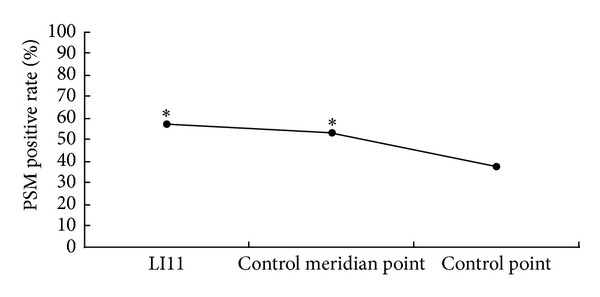
The positive rate of PSM after acupuncture. *Significantly different from control point (*P* < 0.05).

Of the 39 males and 37 females participating in the study, 45 subjects (59.21%) presented positive PSM at LI11, 41 subjects (53.95%) presented positive PSM at the control meridian point, and 29 subjects (38.16%) presented positive PSM at the control point. The results of the Pearson chi-squared test showed that the positive rates of the three stimulation points were not all the same (*χ* ^2^ = 28.298, *P* = 0.000 < 0.05). Further analysis revealed that the difference between LI11 and the control meridian point was not significant (*χ* ^2^ = 0.428, *P* = 0.513 > 0.0167 = 0.05/3). However, the two points showed statistical significance with the control point (*χ* ^2^ = 24.783, *P* = 0.000 < 0.0167 = 0.05/3; *χ* ^2^ = 19.113, *P* = 0.000 < 0.0167 = 0.05/3). The positive PSM rate of LI11 and the control meridian point were obviously higher than that of the control point (*P* < 0.05) (Figure 3).

### 3.2. Comparison of sEMG Amplitude of PSM

**Figure 4:**
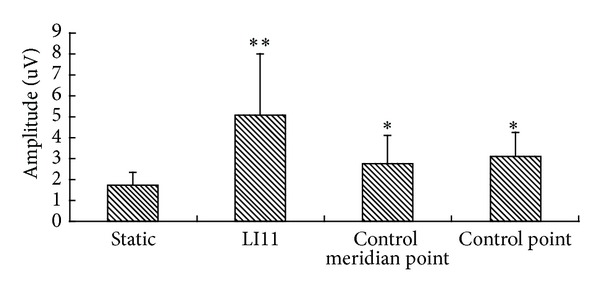
The amplitude variation of surface electromyography after acupuncture. *Significantly different from static condition (*P* < 0.05). **Significantly different from static condition, control meridian point, and control point (*P* < 0.05).

sEMG amplitude of brachioradialis was 1.69 ± 0.63 uV under the static condition. After acupuncture, the amplitude was 5.08 ± 2.93 uV at LI11, 2.77 ± 1.36 uV at the control meridian point, and 3.08 ± 1.18 uV at the control point. There were significant differences in sEMG amplitude at LI11, the control meridian point, and the control point (*P* < 0.05). Acupuncture at LI11 showed significantly larger amplitude than at the control meridian point and the control point (*P* < 0.05). The amplitude of the control meridian point and the control point was significantly larger than at the static condition (*P* < 0.05) (Figure 4).

### 3.3. Duration Comparison of sEMG Activation of PSM

**Figure 5:**
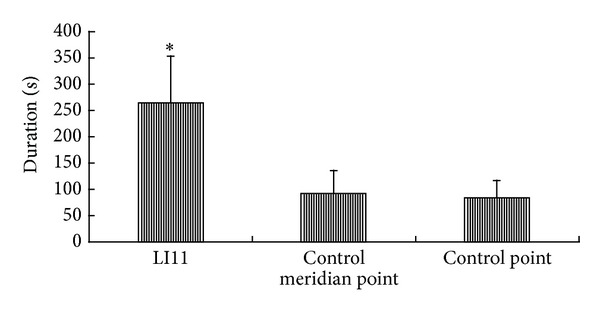
The duration of sEMG activity after acupuncture. *Significantly different from control meridian point and control point (*P* < 0.05).

After acupuncture, when sEMG of brachioradialis returned to the stable baseline, the duration of sEMG was 265 ± 87.87 s at LI11, 91.69 ± 42.98 s at the control meridian point, and 83.31 ± 32.76 s at the control point. The duration showed significant differences at LI11, the control meridian point, and the control point (*P* < 0.05). The duration of LI11 was significantly longer than the control meridian point and the control point (*P* < 0.05) (Figure 5).

## 4. Discussion

PSM in the meridians is a commonly accepted meridian phenomenon and is experienced as a subjective sensation. As Deqi, it played a vital role in the clinical efficacy of acupuncture [2]. Previous research has not shown the anatomical evidence of the meridian. Long considered an essence, the meridian has been studied worldwide to provide biological evidence. Over time, studies of meridians have been performed in the context of neurophysiology-nerve conduction theory, physiology and biochemistry-fluid circulation theory, biology and biological physics field theory, and connective tissue structure theory [10, 11]. Although previous studies presented various evidence of meridians, they failed to clarify the essence of the phenomenon of meridians. sEMG has been widely applied in basic studies observing PSM. Zhu found that the speed of PSM appeared to be synchronized with that of EMG along the meridians. Moreover, the tracks of PSM and EMG appeared in the same place [12, 13]. These studies showed that PSM was not only a subjective sense, but could also be detected by electromyography as an objective indicator.

The present study showed that the PSM of acupuncturing LI11 activated sEMG variations of brachioradialis. The amplitude and duration of sEMG at LI11 were significantly larger than at the control meridian point and the control point. LI11 showed stronger sEMG activation than the control meridian point and the control point on sEMG of brachioradialis (Figures 4 and 5). No significant difference was observed between the control meridian point and control point in the amplitude and the duration of sEMG (Figures 4 and 5), but the positive PSM rate of the control meridian point was significantly higher than that of the control point (Figure 3). LI11 is the He-sea point of large intestine meridian of hand Yangming which is located at the beginning of the brachioradialis, which is in accordance with the running route of the large intestine meridian of hand Yangming. In this study, the control meridian point and the control point were on the muscle belly and the beginning location of brachioradialis, respectively. Therefore, acupuncture caused physical stimulation to the brachioradialis at LI11, the control meridian point, and the control point. After acupuncture, every stimulating point showed significant differences of sEMG activities on brachioradialis, although LI11 showed more obvious sEMG activity. Although the PSM rate was much lower than at the LI11 and the control meridian point, and the control point was not on the large intestine meridian of hand Yangming, the PSM rate of acupuncturing the control point possessed a positive rate of 37.5%. After acupuncture, sEMG amplitude and duration of brachioradialis at LI11 were significantly stronger than at the control meridian point and control point. Moreover, the rate of PSM at LI11 and the control meridian point was higher than at the control point. The results showed that PSM could take place on other parts that are irrelevant to acupoints and meridians and that acupuncturing LI11 and meridian exerted specific effects on sEMG activity of brachioradialis. Thus, PSM could be an objective phenomenon that possesses specificity of acupoints and meridians.

## 5. Conclusion

Although acupuncture was able to activate PSM at all stimulating points, the results suggested that the specificity of sEMG activity of at LI11 was statistically significant, compared with the control meridian point and the control point. In addition, the control meridian point showed greater meridian specificity of sEMG activity than the control point did.

## References

[1] Gao JX, Zhao WB (1995). Discussion on “deqi” of acupuncture and “arrival of Qi” as well as clinical acupuncture. *Journal of Beijing University of Traditional Chinese Medicine*.

[2] Yu SZ, Zhang M, An S (1981). Studies on the phenomenon of latent propagated sensation along the channels. II. Investigation on the lines of LPSC on the twelve main channels. *The American Journal of Chinese Medicine*.

[3] Shang ZD (2011). Essence of meridians and collaterals: circulatory conduction system of bio-electricity of human. *Chinese Acupuncture & Moxibustion*.

[4] Hui KK, Nixon EE, Vangel MG (2007). Characterization of the “deqi” response in acupuncture. *BMC Complementary and Alternative Medicine*.

[5] Li ZR (2007). *Experiment Acupuncture Science*.

[6] Huang T, Yang LJ, Zhang WB (2012). Observation of microvascular perfusion in the hegu (LI4) acupoint area after deqi acupuncture at quchi (LI11) qcupoint using speckle laser blood flow scanning technology. *Evidence-Based Complementary and Alternative Medicine*.

[7] Beissner F, Marzolff I (2012). Investigation of acupuncture sensation patterns under sensory deprivation using a geographic information system. *Evidence-Based Complementary and Alternative Medicine*.

[8] (2008). *WHO Standard Acupuncture Point Locations in the Western Pacific Region*.

[9] Bieuzen F, Lepers R, Vercruyssen F, Hausswirth C, Brisswalter J (2007). Muscle activation during cycling at different cadences: effect of maximal strength capacity. *Journal of Electromyography and Kinesiology*.

[10] Hua P, Lü H, Yuan L, Tang L (2006). Four main schools of thought and analysis in studies of channels and collaterals. *Chineses Acupuncture & Moxibustion*.

[11] Yang HQ, Xie SS, Hu XL, Chen L, Li H (2007). Appearance of human meridian-like structure and acupoints and its time correlation by infrared thermal imaging. *The American Journal of Chinese Medicine*.

[12] Zhu B, Ben H, Xu WD (1997). Secondary excitation in nerve-muscles and propagated sensation along meridians. *Chinese Journal of Basic Medicine in Traditional Chinese Medicine*.

[13] Zhu B, Xu WD, Li YQ (1999). Distribution of electromyography accompanied by propagated sensation along meridians. *Chinese Journal of Basic Medicine in Traditional Chinese Medicine*.

